# Spatial Confinement Engineered Gel Composite Evaporators for Efficient Solar Steam Generation

**DOI:** 10.1002/advs.202407295

**Published:** 2024-09-05

**Authors:** Jun Yan, Tao Cui, Qin Su, Haidi Wu, Wei Xiao, Liping Ye, Suyang Hou, Huaiguo Xue, Yongqian Shi, Longcheng Tang, Pingan Song, Jiefeng Gao

**Affiliations:** ^1^ School of Chemistry and Chemical Engineering Yangzhou University Yangzhou 225002 P. R. China; ^2^ College of Environment and Safety Engineering Fuzhou University Fuzhou 350116 P. R. China; ^3^ College of Material Chemistry and Chemical Engineering Key Laboratory of Organosilicon Chemistry and Material Technology of MoE Key Laboratory of Silicone Materials Technology of Zhejiang Province Hangzhou Normal University Hangzhou 311121 P. R. China; ^4^ Centre for Future Materials University of Southern Queensland, Springfield Campus Springfield QLD 4300 Australia

**Keywords:** carbon aerogel, hydrogel, interfacial evaporation, solar steam generation

## Abstract

Recently, solar‐driven interfacial evaporation (SDIE) has been developed quickly for low‐cost and sustainable seawater desalination, but addressing the conflict between a high evaporation rate and salt rejection during SDIE is still challenging. Here, a spatial confinement strategy is proposed to prepare the gel composite solar evaporator (SCE) by loading one thin hydrogel layer onto the skeleton of a carbon aerogel. The SCE retains the hierarchically porous structure of carbon aerogels with an optimized water supply induced by dual‐driven forces (capillary effects and osmotic pressure) and takes advantage of both aerogels and hydrogels, which can gain energy from air and reduce water enthalpy. The SCE has a high evaporation rate (up to 4.23 kg m^−2^ h^−1^ under one sun) and excellent salt rejection performance and can maintain structural integrity after long‐term evaporation even at high salinities. The SDIE behavior, including heat distribution, water transport, and salt ion distribution, is investigated by combining theoretical simulations and experimental results. This work provides new inspiration and a theoretical basis for the development of high‐performance interfacial evaporators.

## Graphical abstract

The spatially confined hydrogel‐modified interfacial evaporator (SCE) is prepared in this study. Dual‐driven forces for water transportation of SCE including capillary effect and osmotic pressure ensure sufficient water supply for continuous evaporation up to 4.23 kg m^−2^ h^−1^ under one sun and excellent salt rejection performance that it can work in 20 wt.% saltwater stably for the long term.

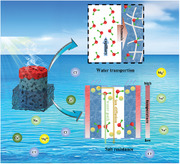

## 1 Introduction

Recently, great achievements have been made in solar‐driven interfacial evaporation (SDIE) technology, as it makes use of both inexhaustible solar energy and abundant seawater resources, showing the potential to solve the growing scarcity of freshwater.^[^ ^1^, ^2^, ^3^, ^4^ ^]^ Much effort has been focused on optimizing thermal localization to maximize the utilization of heat energy,^[^ ^5^, ^6^, ^7^ ^]^ regulating the water supply to ensure the efficiency and sustainability of SDIE,^[^ ^8^, ^9^, ^10^, ^11^ ^]^ reducing the equivalent evaporation enthalpy,^[^ ^12^, ^13^, ^14^ ^]^ and increasing the amount of energy gained from the environment.^[^ ^15^, ^16^ ^]^

Hydrogel composites are now widely used as solar evaporators.^[^ ^17^, ^18^ ^]^ One water molecule typically forms tetrahedral structures with four surrounding water molecules through hydrogen bonds, known as free water (FW).^[^ ^19^ ^]^ The hydrophilic macromolecular chain network of the hydrogel interacts with adjacent water molecules through strong hydrogen bonding, forming bound water (BW). Intermediate water (IW) is present between BW and FW, forming fewer or weaker hydrogen bonds and thus requiring less energy to escape from the water. Peng et al. developed a phase‐separated hydrogel as an interfacial evaporator, which exhibited a high evaporation rate of 2.02 kg m^−2^ h^−1^ in 3.5 wt.% brine, exceeding the theoretical limit of water evaporation.^[^ ^20^ ^]^ Another effective strategy that can significantly increase the evaporation rate is based on the introduction of extra energy, typically by gaining energy from air.^[^ ^21^ ^]^ For a 3D evaporator exposed to a water surface, the temperature of its side surface may be lower than the ambient temperature because of natural evaporation; thus, it can spontaneously absorb heat from the environment. For example, Wu et al. designed an evaporator with a heatsink‐like structure, and the evaporation rate reached as high as 4.10 kg m^−2^ h^−1^.^[^ ^22^ ^]^

Thus, it is reasonable to design solar evaporators that combine hydrogels and aerogels to improve the evaporation rate. Aerogel evaporators are usually exposed to air at a certain height to gain extra energy. Fortunately, water can be quickly pumped to the superhydrophilic evaporator surface through interconnective pores inside the aerogels. In contrast, the water transport speed in hydrogels is usually slower than that in porous aerogels because osmotic pressure‐induced water transport is hindered by polymer chains to some extent. In addition, generating effective convection inside a hydrogel is usually difficult; hence, rapid downward diffusion of salt ions cannot be achieved. Owing to the salting‐out effect, the high concentration of ions at the top causes the aggregation of the polymer molecular chains. As a result, 3D hydrogel evaporators are usually dehydrated, especially when they are exposed to a certain height above the water or in high‐salinity seawater, and this often results in shrinkage and salt precipitation, which is not conducive to long‐term and stable evaporation. Therefore, much effort should be devoted to rational structure design to achieve the synergy of hydrogels and aerogels, i.e., a gel composite evaporator can simultaneously reduce the equivalent water enthalpy and gain energy from the environment while maintaining a powerful water transport capability and salt rejection performance.

**Scheme 1:**
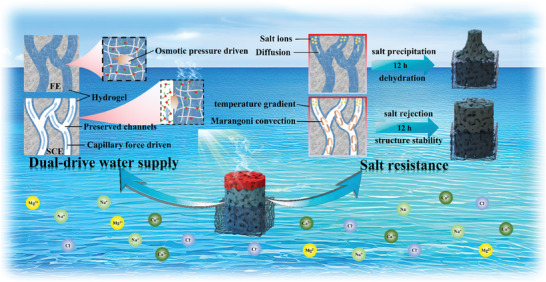
Scheme of the structure of a hydrogel fully filled with an aerogel evaporator (FE) and a spatially confined hydrogel‐modified carbon aerogel interfacial evaporator (SCE).

In this work, we developed a spatial confinement method to load one thin layer of hydrogel on the skeleton of a carbon aerogel for the preparation of gel composite evaporators. As shown in **Scheme** **1**, for a hydrogel fully filled with aerogel evaporator (FE), the transport of water is driven mainly by osmotic pressure. For the spatially confined hydrogel‐modified interfacial evaporator (SCE), the porous structure of the aerogel is almost retained. Water can be first transported upward quickly through preserved channels by capillary action. Then, the sufficient water in the channels diffuses into the hydrogel wrapping the channel wall. The capillary effect and osmotic pressure‐induced water supply, and reduced water enthalpy ensure efficient interfacial evaporation, resulting in a high evaporation rate (up to 4.23 kg m^−2^ h^−1^). When working for a long time in high‐concentration saline water, the FE decreases considerably because of dehydration and salting‐out effects. The SCE can work stably because of Marangoni convection. The heat distribution, water transport and salt ion distribution during interfacial evaporation are investigated by combining theoretical simulations and experimental results. The spatial confinement strategy can effectively address the conflict between the high evaporation rate and salt rejection.

## 2 Results and Discussion

### 2.1 Materials Fabrication and Characterization

**Figure 1:**
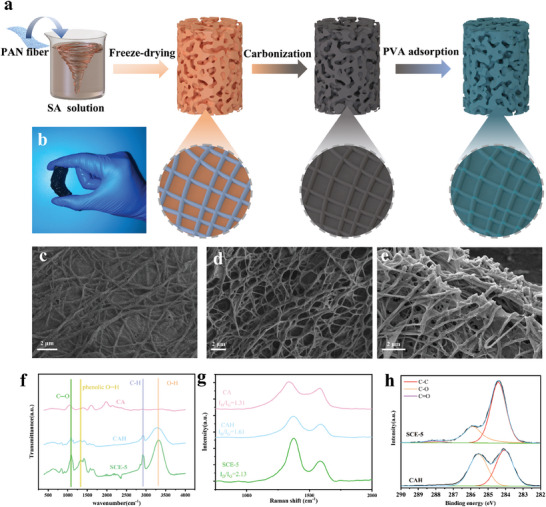
Preparation and microstructures of different hydrogel composites. a) Scheme of the preparation of the SCE. b) Photographs showing the flexibility of SCE‐5. c–e) SEM images of PAN/SA aerogel, CA, and SCE, respectively. f) FTIR spectra and g) Raman shifts of CA, CAH, and SCE. h) High‐resolution X‐ray photoelectron spectroscopy of C1s for CAH and SCE.

The fabrication of the SCE is schematically shown in **Figure** **1**a. The polyacrylonitrile nanofibers were first dispersed in a sodium alginate (SA) solution. Then, the frozen suspension was freeze‐dried to form an aerogel where the nanofibers were embedded into the SA skeleton, forming an interlocking network, as shown in Figure 1c and Figure S1 (Supporting Information). After that, the aerogel was calcined in a nitrogen atmosphere to form a carbon aerogel (CA) with a strong photothermal effect. Figure 1d and Figure S2 (Supporting Information) show that the hierarchically porous structure remained after carbonization. The interlocking nanofiber network structure effectively transferred and dissipated the internal stress, which caused shrinkage and collapse of the carbon skeleton.^[^ ^23^ ^]^ Next, a water/ethanol solution of tannic acid (TA)/polyvinyl alcohol(PVA) was chosen to modify the carbon aerogel. TA is rich in hydroxyl groups and hence can tune the hydrogen bond network of PVA, thereby regulating the state of water molecules. The strong hydrogen bonding between TA and PVA can disrupt the hydration of PVA, resulting in the formation of insoluble flocculent substances (Figure S3a, Supporting Information).^[^ ^24^ ^]^ Hence, ethanol was used to assist in the dissolution of TA in the solution (Figure S3b, Supporting Information). Then, the mixture was quantitatively dropped onto the surface of CA. Due to its superhydrophilicity and hierarchically porous structure, the solution can be spontaneously and rapidly transported to the whole carbon skeleton by capillary forces, forming a thin liquid film. After three freeze‐thaw cycles, gelation occurred to form PVA/TA hydrogels wrapping the skeleton wall of CA. Finally, SCE‐X was obtained after the gel‐modified CA was immersed in deionized water to remove ethanol, where X refers to the mass fraction of PVA in the hydrogel. As shown in Figure 1e and Figure S4 (Supporting Information), the surface of nanofiber is a little rougher. Many carbon nanofibers are separated from each other and attached to the skeleton, indicating that the carbon aerogel is only decorated with one thin layer of hydrogel rather than fully filled with the hydrogel. In contrast, carbon nanofibers are almost invisible and fully buried in the skeleton of FE. Instead, many ultrathin PVA fibrils are observed (Figure S5, Supporting Information), verifying that the skeleton of CA is wrapped by a very thick layer of hydrogel. To further illustrate the difference between the two evaporators, SCE‐5 and FE were pressed hard with a tube containing deionized water dyed with methyl orange (Figure S6, Supporting Information). After 30 s, the water level in the tube on the FE remained unchanged, and no liquid spilled from the FE. On the other hand, the water level in the tube on SCE‐5 gradually decreased, and the red liquid flowed out from the bottom and side of SCE‐5. In addition, the final quality of SCE‐5 is 17–20 times greater than that of the corresponding CA, whereas this value is 40–50 times greater for FE. The results confirm that the spatial confinement strategy enables many large pores to be retained inside SCE‐5, whereas the interconnective pores of CA are blocked by the hydrogel for FE.

Good mechanical performance is a guarantee for durability of SCE‐5.^[^ ^25^, ^26^ ^]^ SCE‐5 shows great flexibility (Figure 1b), and compression tests of a series of evaporators were carried out (Figure S7, Supporting Information). Note that the carbon aerogel is almost incompressible and will collapse when the compression strain is less than 10%, with a maximum compressive stress (MCS) of only 545.9 Pa. When the compression strain was 80%, the MCS of the PVA hydrogel was 50.97 kPa. When TA was introduced, the MCS of the PVA/TA hydrogel reached 197.71 kPa, indicating that strong hydrogen bonds formed between TA and PVA. For the FE, the MCS is as high as 356 kPa. On the other hand, the MCS of SCE increases with increasing PVA concentration, and the internal stress is deconcentrated by the deformation of the pores and the dense hydrogel layer. SCE‐5 has an MCS of 101.38 kPa, which is much larger than that of CA but smaller than that of the PVA/TA hydrogel and FE with solid structures. These results verify that the thin hydrogel layer significantly enhances not only the strength but also the flexibility of the carbon aerogel, making the SCE evaporator mechanically robust for interfacial evaporation. Furthermore, spatial confinement of the hydrogel preserves many interconnected pores inside the SCE for continuous water transportation and vapor escape during interfacial evaporation.

Figure 1f shows the FTIR spectra of the dried CA, PVA hydrogel‐modified carbon aerogel (CAH), and SCE‐5. Note that CAH and SCE were freeze‐dried to eliminate the effects of water. The three samples all show C─O stretching vibration peaks at ≈1100 and 3000 cm^−1^. For CAH and SCE, the stretching vibrations of C─H and O─H are at ≈2918 and ≈3300 cm^−1^, respectively, indicating that the PVA hydrogel was successfully loaded onto CA.^[^ ^27^ ^]^ Only the SCE exhibited a phenolic O─H peak at ≈1320 cm^−1^, indicating the successful introduction of TA. The O─H peak of SCE shows a blueshift of 38 cm^−1^, which may be caused by TA‐induced hydrogen bond reconstruction.^[^ ^24^ ^]^ Figure 1g shows the Raman spectra of CA, CAH, and SCE. The intensity ratio of the D peak at ≈1350 cm^−1^ to the G peak at ≈1680 cm^−1^ (***I_D_***/***I_G_***) of CA is 1.31, indicating a relatively low degree of graphitization caused by the low calcination temperature (500 °C). Therefore, many heteroatoms and defects are retained, and they are responsible for the superhydrophilicity of CA to a certain extent. After being modified by hydrogels, ***I_D_***/***I_G_*** of CAH and SCE increased to 1.61 and 2.63, respectively, because of the interfacial interaction of PVA macromolecular chains and the residual functional groups on the carbon skeleton during freeze‐thaw cycles. The full XPS spectra for CAH and SCE are shown in Figure S8 (Supporting Information). All the samples contain four elements, i.e., Na, C, N, and O. The high‐resolution C 1s spectrum is shown in Figure 1h, and SCE has an extra peak at 288 eV, which is ascribed to the C═O of TA.

### 2.2 Photothermal Performance of SCEs

Excellent photothermal conversion performance is extremely important for interfacial evaporators.^[^ ^28^ ^]^ The absorption of light from 300 to 2500 nm of different evaporators was first measured. The average absorption (α) is as follows:

(1) $$\alpha =\frac{\int_{300}^{2500}I\left(\lambda \right)\cdot A\left(\lambda \right)d\lambda }{\int_{300}^{2500}I\left(\lambda \right)d\lambda }$$

where *I*(λ) and *A*(λ) are the light intensity and absorption at the wavelength of λ, respectively.

**Figure 2:**
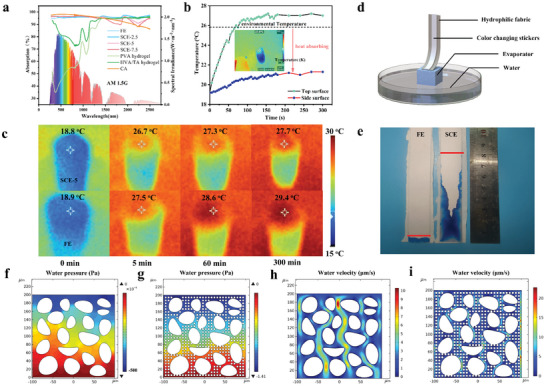
Photothermal conversion and water transportation behavior of the evaporators. a) UV‒vis–NIR spectra of CAs, hydrogels, and their composites. b) Top and side surface temperature variations of SCE‐5 under one sun during interfacial evaporation. The insets are the corresponding infrared images. c) Infrared thermographic images of SCE‐5 and FE irradiated under one sun for 300 min. d) Schematic of the device and e) photograph showing the water supply of the SCE. Simulated water pressure distributions inside f) SCE‐5 and g) the FE during evaporation. Simulated water velocity distribution in the internal channels of h) SCE‐5 and i) FE. Note that the simulation was conducted using COMSOL Multiphysics 5.5.

**Figure** **2**a shows that the PVA hydrogel has the lowest light absorption (43.4%) and can absorb near‐infrared light strongly, which is attributed to the stretching vibration of the O─H of water molecules; however, the white surface limits its absorption of visible light, which contains most of the solar energy. With the introduction of TA, the PVA/TA hydrogel turns pale yellow and hence has greater absorption of visible light. In addition, owing to the π→π^*^ interactions of the aromatic electrons of TA, the PVA/TA hydrogel also has high absorption in the ultraviolet band. The light absorption of CA is considerably enhanced and can reach 97.2% in the full spectrum range from 300 to 2500 nm because of the hierarchical porous structure and carbon skeleton. On the other hand, the absorption of the SCEs and FE slightly decreased in the UV‒vis region. Notably, the light absorption of the SCEs is slightly greater than that of the FE because the SCEs retain the hierarchically porous structure of the original aerogel and capture photons through multiple scattering of light.

Figure 2b shows the photothermal conversion behavior of SCE‐5. The cylindrical evaporator floats on the water with the assistance of one piece of Pearl cotton foam. The bottom of the evaporator was immersed in water, and the height above the water was 4 cm. The temperature of the evaporator is only 18.8 °C with no irradiation, which is much lower than the environmental temperature (25.8 °C) because natural water evaporation in the dark takes away energy. Under irradiation at 1000 W m^−2^ (one sun), the top surface temperature rises rapidly in the first 100 s and then gradually stabilizes at an equilibrium temperature of ≈27.0 °C. On the other hand, the side surface temperature increases by only 2.5 °C, up to 21.3 °C, which is still lower than the temperature of the environment and bulk water, leading to the extra energy input to enhance interfacial evaporation. The temperature distribution was simulated using COMSOL Multiphysics 5.5 (Figure S9, Supporting Information). The simulation revealed that the highest and lowest temperatures are located at the center of the top surface (26 °C) and the lower part of the side surface (22.1 °C), which is consistent with the results in Figure 2b. Consequently, SCE can exceed the limits of the theoretical evaporation rate and efficiency. The infrared thermal images in Figure 2c show the temperature evolution of SCE‐5 and FE under one sun for 5 h. The initial temperature difference between the two evaporators is only 0.1 °C, indicating their similar dark evaporation performance. However, as the illumination starts, the temperature of the FE is higher than that of the SCE‐5. The generated heat by sunlight is generally divided into three parts, namely heat loss, sensible heat which leads to an increase in the surface temperature of the evaporator, and latent heat which converts water from liquid to vapor. This indicates that the water supply of the FE is insufficient. Thus less heat is converted into latent heat for interfacial evaporation, and correspondingly, more heat is used to increase the surface temperature. The initial CA has an interconnected macroporous structure and superhydrophilicity, ensuring fast water transport. Fortunately, SCEs still retain this porous structure to maintain a powerful water transport capacity, while a hydrogel fully filled in CA weakens water transport. To illustrate the effect of the spatially confined structure of the hydrogel on water transport, the evaporator was placed in water (Figure 2d), with the top surface exposed to air. A superhydrophilic fabric was connected to the top surface of the evaporator, and a color indicator sticker was pasted on the fabric. Water infiltrated the fabric through the evaporator and then was transported upward by capillary forces. The water in the fabric subsequently diffused into the sticker and caused the sticker to change color. After 1 min, the water was pumped to a height of 8 cm through the SCE‐5 but to less than 1 cm through the FE, indicating the advantage of a spatially confined structure (Figure 2e). In Figure 2f–i, two comparative simulations are set up. One simulated object is based on a micron‐scale large pore structure corresponding to SCE‐5, whereas the other is filled with many small circles corresponding to the FE. Suppose that the top surface of two simulated objects has the same evaporation rate at any given time, and the water pressure and water velocity inside the objects are investigated. There are two main driving forces for upward water transportation:

(2) $$P_{o}=cRT$$

(3) $$P_{c}=\frac{2\lambda \;cos\theta }{r}$$

where *P_o_* (Pa) is the osmotic pressure; *c* (mol L^−1^) is the concentration of water molecules; *R* (8.314 J mol^−1^ K^−1^) is the gas constant; and *T* is the temperature of the water. *P_c_* (Pa) and λ (N m^−1^) refer to the capillary force and surface tension of water, respectively; θ and *r* (m) represent the contact angle of water and the capillary radius, respectively. There will be a negative pressure difference in the direction of water flow, which is positively correlated with the water velocity. When the total driving force of water is greater than the sum of the negative pressure and gravity of water, a stable water supply is formed. Otherwise, insufficient water transport will occur. A smaller water channel results in a higher water transporting velocity and a higher negative water pressure. SCE‐5 can pump water with a dual‐driven force and only needs to generate a water velocity of ≈5 µm s^−1^ and overcome a negative water pressure of 0.25 Pa mm^−1^. (Figure 2f,h) The FE generates a water velocity of ≈12 µm s^−1^ and overcomes the water pressure of 7.5 Pa mm^−1^ (Figure 2g,i), which is ≈30 times higher than that of SCE‐5. Unfortunately, the pores are fully filled with PVA hydrogels for FE, and water molecules in the hydrogels are trapped by PVA chains because of strong hydrogen bonding, which limits water transportation and evaporation to some extent. It is clear that spatial confinement enables porous channels to endow SCEs with powerful water transport performance, making it possible for the evaporator to be exposed to the water surface at a large height to absorb a large amount of heat from the environment.

### 2.3 Evaporation Performance of SCEs

**Figure 3:**
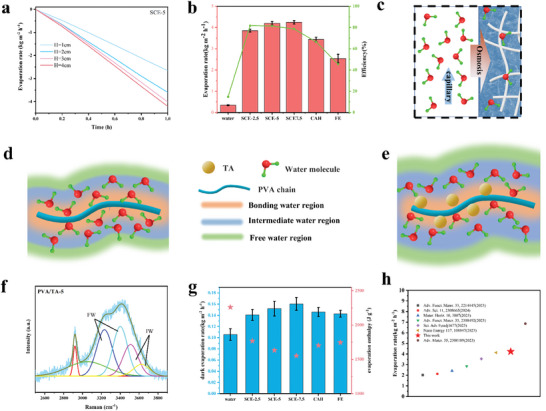
Interfacial evaporation performance of the SCE. a) Mass loss of water with time under one sun using SCE‐5 evaporators exposed to the water surface at different heights. b) Evaporation rates and evaporation efficiencies for blank water, SCE‐2.5, SCE‐5, SCE‐7.5, CAH, and FE where the mass fraction of PVA in the hydrogel is 2.5, 5, and 7.5 wt.% for SCE‐2.5, SCE‐5, and SCE‐7.5, respectively. c) Scheme of the dual‐driven force during evaporation. d,e) Schemes of the water molecule state in CAH and SCE, respectively. f) Fitting curves of the Raman shift of water in the PVA/TA‐5 hydrogels from 2500 to 4000 cm^−1^. g) Evaporation rates and enthalpies of different evaporators measured by dark evaporation. h) Comparison of evaporation rates between SCE‐5 and other hydrogel‐based evaporators.

As shown in **Figure** **3**a, the height of the evaporator above the water was changed to investigate its influence on the evaporation performance. With increasing height, the exposed side area becomes larger, and the evaporator tends to gain more energy from the air, hence enhancing evaporation. Notably, the enhancing effect of the exposed height on the evaporation rate gradually decreases because the higher the height is, the greater the gravitational potential energy that water transportation must overcome. In the subsequent tests, the height of all the interfacial evaporators above the water surface was set to 4 cm. The average evaporation rates and efficiencies of the different evaporators in 3.5 wt.% seawater under one sun for 10 h are shown in Figure 3b. The FE has a low evaporation rate of 2.54 kg m^−2^ h^−1^ because the inferior water transport is driven mainly by osmotic pressure. Owing to the preserved porous structure and superhydrophilicity of SCEs, water can be transported quickly through dual‐driven forces, resulting in high evaporation rates (up to 4.23 kg m^−2^ h^−1^). This dual‐driven mode is shown in Figure 3c. Here, the water in the evaporation system can be divided into two forms, namely, the water in the spatially confined hydrogel (HW) and the water in the porous channel (CW). When energy is input, the activated HW is preferentially evaporated, resulting in an osmotic pressure difference between the HW and the CW; thus, the CW is absorbed into the thin hydrogel layer and transformed into an HW. On the other hand, CWs can be quickly supplied from bottom bulk water through capillary forces. This “bulk water–CW–HW–vapor” mode under dual‐driving forces shortens the mass transfer path, ensuring rapid and stable evaporation performance. In the “bulk water–HW–vapor” mode for FE, water diffuses through a dense polymer network with high diffusion resistance, making it difficult to achieve effective evaporation. Additionally, HW has a reduced evaporation enthalpy; hence, SCE‐5 has a greater evaporation rate (4.23 kg m^−2^ h^−1^) than CA does (3.82 kg m^−2^ h^−1^), as shown in Figure S10 (Supporting Information). When the concentration of PVA increases from 2.5 to 5 wt.%, the evaporation rate increases from 3.85 to 4.23 kg m^−2^ h^−1^, while the evaporation rate of SCE‐7.5 is similar to that of SCE‐5. In addition, the introduction of TA increases the evaporation rate from 3.72 kg m^−2^ h^−1^ for CAH‐5 to 4.23 kg m^−2^ h^−1^ for SCE‐5, indicating that TA can reconstruct the hydrogen bond network between water molecules and PVA inside the evaporator. As shown in Figure 3d, water molecules can be divided into FW, IW, and BW depending on the strength or number of hydrogen bonds around them. The water molecules close to the PVA chain form BW with strong hydrogen bonding. BW is not involved in evaporation. In the outer layer of BW, IW with weak or few hydrogen bonds is formed, which easily escapes from the bulk water in the form of molecular clusters. Therefore, the average enthalpy of evaporation of water molecules in the interfacial evaporator depends mainly on the ratio of FW to IW. As shown in Figure 3e, TA can replace some BW and form stronger hydrogen bonds with PVA molecules, and the replaced BW is transformed into IW or FW. Moreover, TA has abundant hydroxyl groups, which can provide more active sites and form more IW, thus further reducing the enthalpy of evaporation. Figure 3f shows the Raman spectrum of water inside PVA/TA‐5 in the range of 2500–4000 cm^−1^. The peak at ≈2900 cm^−1^ is assigned to the stretching vibration of the C─H bonds of PVA. The two peaks at 3230 and 3400 cm^−1^ are associated with the symmetric stretching vibration of free water, whereas the other two peaks at 3514 and 3630 cm^−1^ correspond to the asymmetric stretching vibrations of intermediate water. The Raman fitting curves of water in the hydrogels are shown in Figure S11 (Supporting Information). The ratios of FW to IW were 2.00, 2.15, 2.28, and 2.84 for the PVA/TA‐7.5, PVA/TA‐5, PVA/TA‐2.5 and PVA hydrogels, respectively. In addition, dark evaporation experiments were carried out to further quantitatively evaluate the average evaporation enthalpy of the water inside each evaporator. The detailed experiment is shown in ES1 (Supporting Information). The average enthalpy can be calculated as follows:

(4) $$H_{0}\;m_{0}=H_{i}\;m_{i}$$

where *H*_0_ represents the enthalpy of pure water, *m*_0_ represents the mass loss of water without the use of evaporators during dark evaporation, *m_i_* represents the mass loss of water via different evaporators, and *H_i_* represents the enthalpy of the water inside the corresponding evaporators. The calculated average enthalpy of evaporation is shown in Figure 3g. The water enthalpy decreases with increasing PVA concentration, whereas TA can further optimize the evaporation enthalpy of water. LiCl was used to further verify the water state during interfacial evaporation because it can bond with water clusters in the solution.^[^ ^12^ ^]^ SCE‐5 was used as the evaporator to vaporize 1000 mg L^−1^ of LiCl solution, and the control group experiments were conducted by evaporating the LiCl solution without using an evaporator. The Li^+^ concentration in the collected condensate water from the interfacial evaporation of SCE‐5 reached 14.3 mg L^−1^ (Figure S12, Supporting Information), whereas the value of the control group was only 0.25 mg L^−1^, indicating that SCE‐5 enables the evaporation of water molecules in the form of clusters. The spatial confinement of engineered gel composite solar evaporators results in a high interfacial evaporation rate exceeding that of most other reported hydrogel evaporators (Figure 3h).^[^ ^20^, ^29^, ^30^, ^31^, ^32^, ^33^, ^34^ ^]^

**Figure 4:**
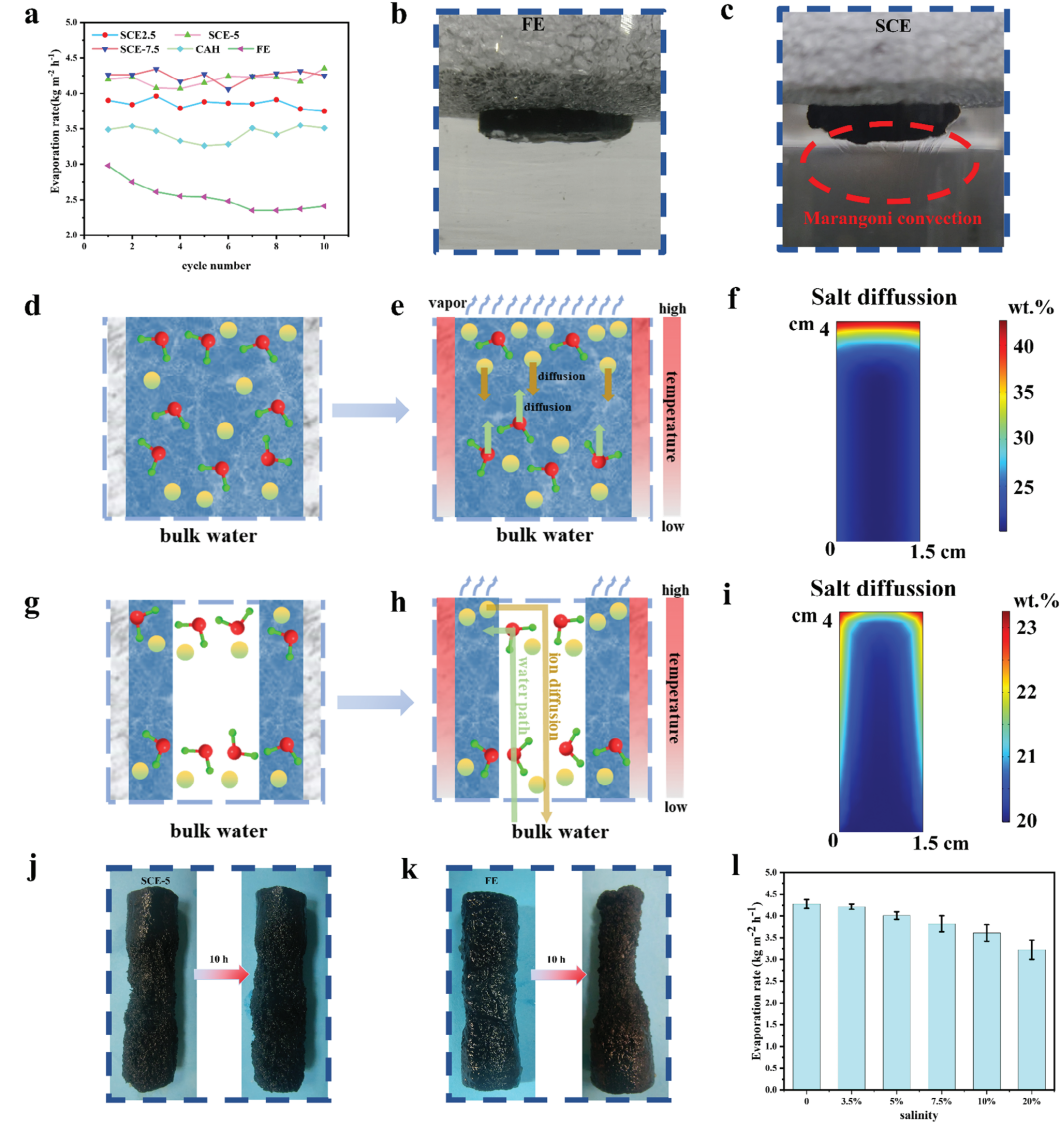
Salt rejection performance and durability of the SCE. a) Evaporation rates of different interfacial evaporators within 10 h in 3.5 wt.% saltwater. b,c) Photos of FE and SCE‐5 during evaporation, respectively. d,e,g,h) Schemes of the salt resistance of FE and SCE, respectively. f,i) Simulated salt diffusion paths in FE and SCE‐5, respectively. j,k) Images of SCE‐5 and FE before and after 10 h of irradiation in 20 wt.% saltwater, respectively. l) Evaporation rates of SCE‐5 in salt water with different salinities.

The long‐term evaporation test results of different interfacial evaporators in 3.5 wt.% salt water are shown in **Figure** **4**a. The initial evaporation rate of the FE was ≈3.05 kg m^−2^ h^−1^. However, as the irradiation time increases, the evaporation rate gradually decreases, which is caused by an insufficient water supply and the salting‐out effect. All SCEs show stable evaporation performance with almost no change in the evaporation rate. The concentrated ions at the evaporation interface can freely diffuse from the thin hydrogel layer to the water channel. Moreover, because the temperature gradient induced a tension gradient, Marangoni convection occurred in the channels of the SCE, resulting in rapid mass transfer of ions. As shown in Figure 4b,c, Marangoni convection is clearly observed below the SCE but is absent for the FE during evaporation. The schemes of salt diffusion for the FE are shown in Figure 4d,e. As water evaporates, salt ions concentrate on the evaporation surface and form a concentration gradient with the bulk water at the bottom, thus spontaneously diffusing downward. However, according to hydrodynamic theory, polymer networks can hinder the diffusion of ions.^[^ ^35^, ^36^ ^]^ Due to the limited Marangoni convection, the rapid diffusion of ions cannot be realized by relying solely on a concentration gradient, resulting in an increased salt concentration. As a result, salt will accumulate on the top surface and cause the FE to shrink. The salt distribution inside the FE and SCE during interfacial evaporation is simulated (Figure 4f,i), and FE results in a high salt concentration of over 40 wt.%, which exceeds the solubility of NaCl. In fact, during the actual process, the top surface dried and contracted, making it impossible to evaporate continuously. SCE shows good salt resistance because of the optimized water path. As shown in Figure 4g,h, as the aerogel wall transformed light to heat, the water in the hydrogel close to the hot wall preferentially evaporated because of its low evaporation enthalpy. Owing to the sharp increase in the ion concentration in the top hydrogel layer, osmotic pressure is generated. There are two possible ways for ions to diffuse. One way is to diffuse to the bottom of the hydrogel layer, and the other way is to diffuse to the channel outside the hydrogel layer. Owing to sufficient Marangoni convection in the water channel, the accumulated salt ions can be quickly transported to the bulk water at the bottom, while the movement of ions in the hydrogel will be hindered by polymer chains and a dense pore structure; thus, salt ions preferentially diffuse into the water channel. The simulation results (Figure 4i) indicate that SCE‐5 can maintain a relative low concentration of ≈23 wt.% after working in 20 wt.% salt water for 12 h. The optimized water path ensures continuous solar steam generation. As shown in Figure 4j,k, SCE‐5 can maintain its initial structure after working in 20 wt.% salt water. In contrast, the upper part of the FE severely contracted due to the slow water supply and salt‐outing effects. Moreover, the shrunk evaporator densifies the polymer network in the hydrogel, further weakening its water supply capacity and exacerbating deformation. Figure 4l shows the excellent interfacial evaporation performance of SCE‐5 in brine at different concentrations. When the concentration increases from 3.5 to 20 wt.%, the evaporation rate of SCE‐5 decreases slightly but remains at 3.25 kg m^−2^ h^−1^, indicating its high practical application potential.

**Figure 5:**
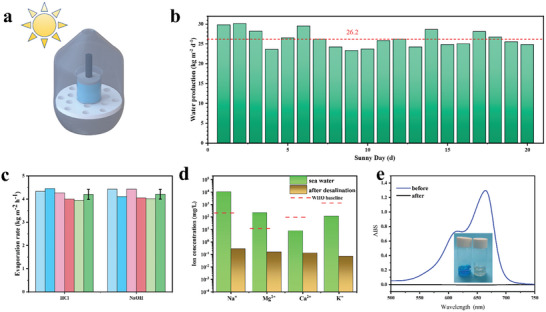
Interfacial evaporation performance of SCE‐5 during practical applications. a) Scheme of the device for outdoor desalination. b) Water yield of 20 sunny days. c) Evaporation rate in acidic and alkaline solutions. d) Concentrations of several different metal ions in seawater before and after desalination. e) Ultraviolet absorption curves of methylene blue solution and condensed water. The insert: photo of the solution and condensate water.

To explore the practical applications of SCEs, we designed a set of outdoor evaporation devices. As shown in **Figure** **5**a, the SCE was placed in a container for SDIE, and the generated water vapor liquefied into droplets at the top of the container and flowed along the wall to the bottom. The device was placed on the roof of a student dormitory in Yangzhou, and mass loss was recorded daily from July to August. As shown in Figure 5b, during the 20 sunny days, the average daily water production reached 26.2 kg m^−2^ d^−1^. Note that owing to the closed environment and sunlight loss at the top of the container, the actual outdoor evaporation rate is lower than the experimental evaporation rate when the simulative light source is used. And the water collection efficiency can be improved by reducing the condensation surface temperature, changing air convection, etc.^[^ ^37^, ^38^ ^]^ In addition, Figure 5c shows that the evaporation rate of SCE‐5 remains stable during continuous desalination for 5 h in acidic (pH = 3) or alkaline (pH = 11) solutions, and the average evaporation rates are 4.23 and 4.21 kg m^−2^ h^−1^ respectively. There are four main metal ions in seawater, namely, Na^+^, Mg^2+^, Ca^+^, and K^2+^. The concentrations of these four ions before and after desalination were measured via an inductively coupled plasma spectrometer. Figure 5d shows that all four ions decrease to less than 10 mg L^−1^, and the concentration of Na^+^ decreases by three orders of magnitude, meeting the WHO standard for drinking water. As shown in Figure 5e, when SCE‐5 is used to treat 10 mg L^−1^ methylene blue solution, the collected condensed water is colorless and transparent and shows almost no absorption in the UV‒vis region, which confirms that the condensate does not contain any dye pollutants, indicating its potential to purify organic polluted wastewater.

## 3 Conclusion

In conclusion, we developed a simple spatial confinement method to load one thin hydrogel layer on the skeleton of a carbon aerogel as an interfacial evaporator. By combining the advantages of hydrogels and aerogels, SCE evaporators can optimize energy management by absorbing extra energy from the environment while simultaneously reducing the water evaporation enthalpy. Compared with CA, SCEs exhibit enhanced compressive strength and flexibility. In addition, benefiting from dual‐driven forces, the spatially confined structure endows the SCE with an optimized water path. This material exhibits high light absorption and strong photothermal conversion from 300 to 2500 nm. When used as solar‐interfacial evaporators, SCEs have high evaporation rates (up to 4.23 kg m^−2^ h^−1^) with excellent salt rejection performance and can work stably in salt water due to Marangoni convection. Finite element simulation reveals the influence of spatially confined structures on fluid flow and salt distribution. This work provides a new perspective for structural design to develop high‐performance interfacial solar evaporators.

## Conflict of Interest

The authors declare no conflict of interest.

## Supporting information

Supporting Information

## Data Availability

The data that support the findings of this study are available in the supplementary material of this article.
