# Supplementary material for: Spatial Confinement Engineered Gel Composite Evaporators for Efficient Solar Steam Generation
Source: Adv Sci (Weinh). 2024 Sep 5;11(41):2407295. doi: 10.1002/advs.202407295 (PMC11538639; doi:10.1002/advs.202407295)
Supplement: Supplementary file 1 — Supporting Information [file ADVS-11-2407295-s001.docx]

**Spatial confinement engineered gel composite evaporators for efficient solar steam generation**

Jun Yan, Tao Cui, Qin Su, Haidi Wu, Wei Xiao, Liping Ye, Suyang Hou, Huaiguo Xue*, Yongqian Shi, Longcheng Tang, Pingan Song, Jiefeng Gao*

J. Yan, T. Cui, Q. Su, H. Wu, W. Xiao, L. Ye, S. Hou, H. Xue, J. Gao

School of Chemistry and Chemical Engineering

Yangzhou University,

Yangzhou 225002, PR China

Email: [chhgxue@yzu.edu.cn](mailto:chhgxue@yzu.edu.cn); [jfgao@yzu.edu.cn](mailto:jfgao@yzu.edu.cn).

Y. Shi

College of Environment and Safety Engineering

Fuzhou University

Fuzhou 350116, China

L. Tang

College of Material, Chemistry and Chemical Engineering

Key Laboratory of Organosilicon Chemistry and Material Technology of MoE

Key Laboratory of Silicone Materials Technology of Zhejiang Province

Hangzhou Normal University

Hangzhou 311121, China

P. Song

Centre for Future Materials

University of Southern Queensland

Springfield Campus, QLD 4300, Australia

**S1. Methods**

**S1.1 Materials**

Polyvinyl alcohol (PVA, 1799) powders were provided by Shanghai Macklin Biochemical Co., Ltd. Sodium alginate (SA, AR, 90%), and polyacrylonitrile (PAN, average M_w_: 149000 - 151000) and tannic acid (TA, AR, 90%) were obtained from Shanghai Aladdin Biochemical Technology Co., Ltd, respectively. N, N-Dimethylformamide (DMF, AR) was received from Sinopharm Chemical Reagent Co., Ltd.

**S1.2 Fabrication of PAN nanofiber membrane**

The fabrication of PAN nanofiber membrane can be found in our previous work.^1^ Typically, a certain PAN powders were dissolved in DMF at 65 °C for 12 h to obtain a homogeneous solution with the PAN concentration of 13 wt.%. Then, the solution underwent electrospinning under a voltage of 18 kV and a feeding rate of 2 ml h^-1^. The obtained membrane was dried in an oven at 60 ℃ for 12 h to remove the residual solvent.

**S1.3 Fabrication of carbon aerogel (CA)**

A certain mass of PAN nanofiber membrane was cut into many pieces and then added into 0.5 wt.% sodium alginate solution, and the mass ratio of PAN to SA was 1:1. Then the mixture was poured into a homogenizer for dispersion (10 minutes) with the razor rotation speed of 32, 000 rpm. The obtained white dispersion was poured into a mould to freeze at -24 °C for 12 h and then freeze-dried for 48 h to obtain the aerogel. The carbon aerogel (CA) was obtained by calcination of the aerogel at 500 °C for 2 h under a nitrogen atmosphere.

**S1.4 Fabrication of PVA hydrogel and PVA/TA hydrogel**

0.1 g TA, 10 g ethanol, and 5 g PVA were mixed with water in a beaker. After stirring at 90 ℃ for 5 h, a transparent yellow solution (110 g) was obtained (solution A). Then the solution was poured into the mould and frozen at -20 ℃ for 8 h followed by thawing at room temperature for another 8 h. After three repetitions, the composite was placed in deionized water for 48 h to remove the ethanol to obtain the PVA/TA hydrogel. The PVA hydrogel is prepared using the same method without adding TA.

**S1.5 Fabrication of hydrogel modified carbon aerogel composites.**

A certain amount of solution A was dripped onto the surface of CA, and the mass ratio of the solution to CA is controlled at 20 : 1. After standing for 5 minutes, the aerogels containing the solution were frozen at -20 ℃ for 8 h, followed by thawing at room temperature for another 8 h. After three repetitions, the gel composites were placed in deionized water for 48 h to remove the ethanol. The obtained spatially confined PVA/TA hydrogel modified carbon aerogel evaporator is named SCE-X, where X represents the concentration of PVA. For comparison, the spatially confined PVA hydrogel modified carbon aerogel (represented by CAH) was also prepared by using the PVA solution instead of PVA/TA solution. For fabrication of carbon aerogel fully filled with the hydrogel (FE), the aerogel was directly immersed into the solution A, and the subsequent procedures is the same as that for preparation of SCE.

**S2. Evaporation test**

The interfacial evaporator floated on the water with the assistance of a piece of Pearl cotton inside the beaker. The whole device was placed on a digital balance that can automatically send data to the computer every 5 s. A simulated light source was placed above the device and the light intensity at the top of the evaporator was adjusted to 1000 W m^-2^. The evaporation rate $(\nu)$ and efficiency $(\eta$) are calculated by EP (1-3):

|  | $\nu=\frac{dm}{S_{top} \cdot dt}$ | (1) |
| --- | --- | --- |
|  | $E_{in}=S_{top}\cdot P_{sol}+\varepsilon\sigma\left( T_{env}^{4}-T_{side}^{4} \right)\cdot S_{side}+h\cdot(T_{env}-T_{side}) \cdot S_{side}$ | (2) |
|  | $\eta=\frac{m H_{evap}}{E_{in}}$ | (3) |

where $m$ is the mass change of the seawater. $t$ is the evaporation time. $S_{top}$ and $S_{side}$ are the top surface aera and side surface aera, respectively. $T_{env}$ and $T_{side}$ are temperature of environment and side surface, respectively. $P_{sol}$ is the light intensity, $\varepsilon$ is the emission of the evaporator, $\sigma$ is the Stefan-Boltzmann constant, $h$ is convection heat transfer coefficient $m$ is the mass change of the seawater, and $H_{evap}$ is the evaporation enthalpy of water in evaporator.

**S3 Simulation Part**

**S3.1. Simulation of water velocity**

To simulate the water velocity and pressure in microchannels inside evaporators. A constant solar irradiation of 1 kW m^−2^ was applied to the top surface for driving evaporation. The water flux entered the evaporator equals to the escaped water flux caused by evaporation.

|  | $\rho\left( u\times\nabla\right)u=\nabla\times\left( -PI+\mu\left( \nabla u+\left( \nabla u \right)^{T} \right) \right)+F+\rho g$ | (4) |
| --- | --- | --- |
|  | $\rho\nabla\times u=0$ | (5) |

where the 𝜌 and 𝜇 are the mass density and viscosity of water. $u$, $P$, and *T* represent the fluid flow speed, pressure, and temperature of water, respectively. *I* and *F* are the second order unit tensor and gravity of water, respectively.

**S3.2. Simulation of heat distribution**

To simulate the salt and heat distribution in evaporators, a mass flux of NaCl (J_evap_) was applied on the evaporation interface.

| $J_{evap}=\frac{m\cdot c}{\rho}$ | (6) |
| --- | --- |
| $m=-k_{m}\frac{M}{R}(\frac{P_{w}^{v,sat}}{T}-H_{R}\frac{P_{e}^{v,sat}}{T_{e}})$ | (7) |
| $P_{x}^{v,sat}=P_{0}exp(\frac{h_{evap}}{R}M(\frac{1}{T_{0}}-\frac{1}{T}))$ | (8) |

where ***M*** represents the molar mass of water (18 × 10^−3^ kg mol^−1^), ***R*** is the universal gas constant (8.314 J mol^−1^ K^−1^), $P_{w}^{v,sat}$ and $P_{e}^{v,sat}$are the pressures of water vapor at the surface temperature (***T***) and at the environment temperature ($T_{e}$), respectively, H_R_ is the relative humidity, $k_{m}$ is the convective mass transfer coefficient, $P_{0}$ is the standard pressure and $T_{0}$ is the boiling point of water. ^2^

**ES1. Estimation of Equivalent Evaporation Enthalpy**

To obtain the vaporization enthalpy, a control experiment was designed to estimate the

evaporation enthalpy. The water and evaporators with same surface area were set in a closed container together under a temperature of 30 ºC. The vaporization enthalpy of water in evaporators can be estimated vaporizing the water with identical power input (*U_in_*):

${U_{in}=H}_{0} m_{0}=H_{i} m_{i}$ (9)

where $H_{0}$ represents the enthalpy of pure water, $m_{0}$ is the mass loss of water without using any evaporators during dark evaporation. $m_{i}$ is the mass loss of water using different evaporators and $H_{i}$ is the enthalpy of the water inside corresponding evaporators.

**Figures**


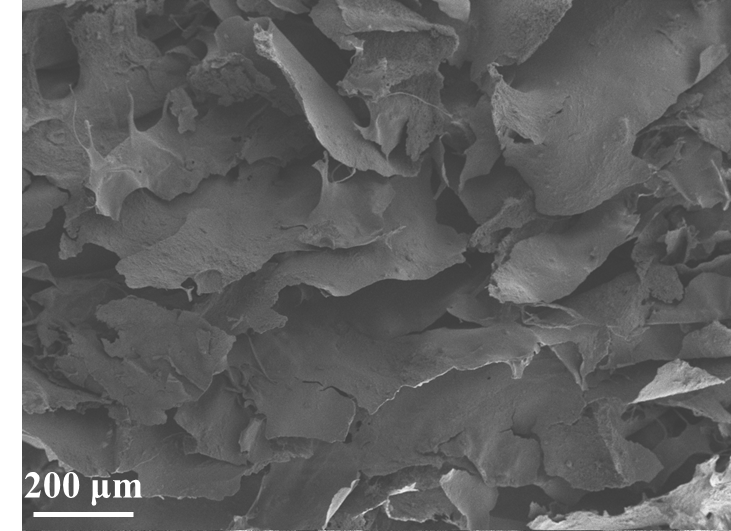


Fig. S1 SEM images of SA aerogel


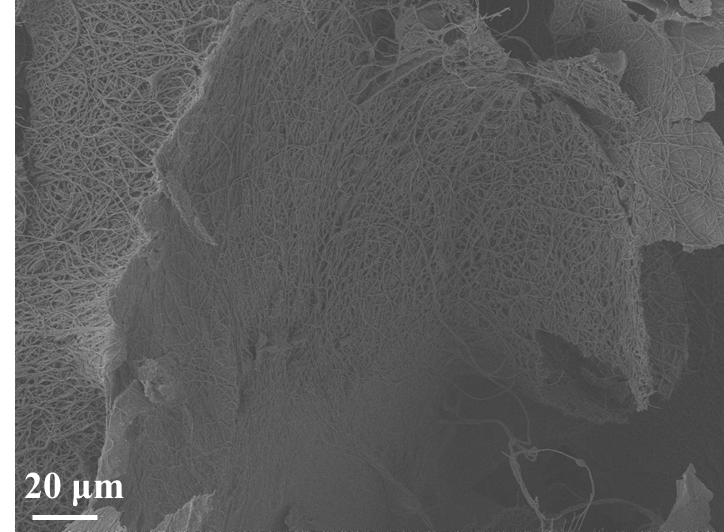


Fig. S2 SEM images of CA


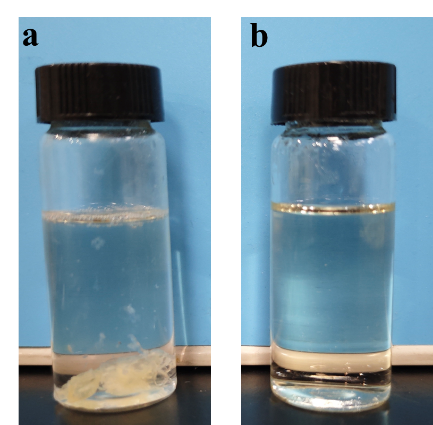


Fig. S3 images of PVA/TA dissolved in water without and with ethanol.


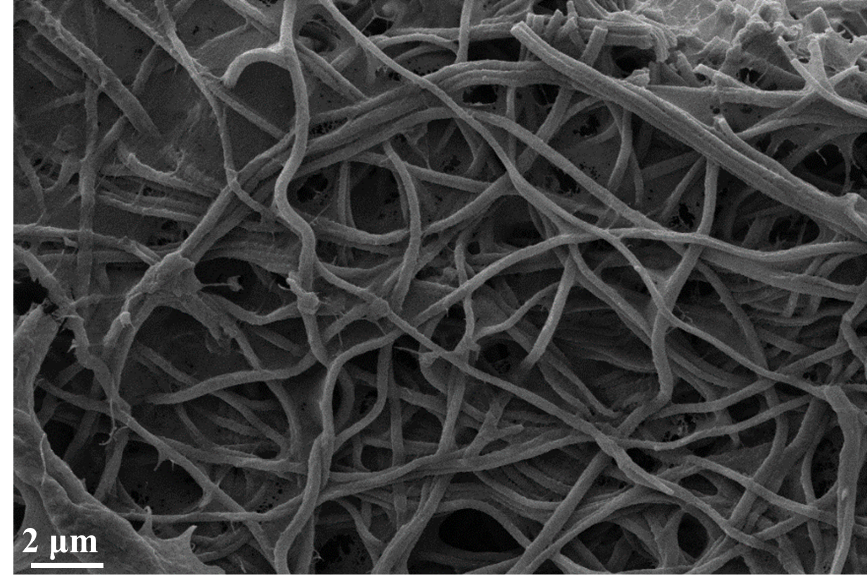


Fig. S4 SEM images of SCE-5


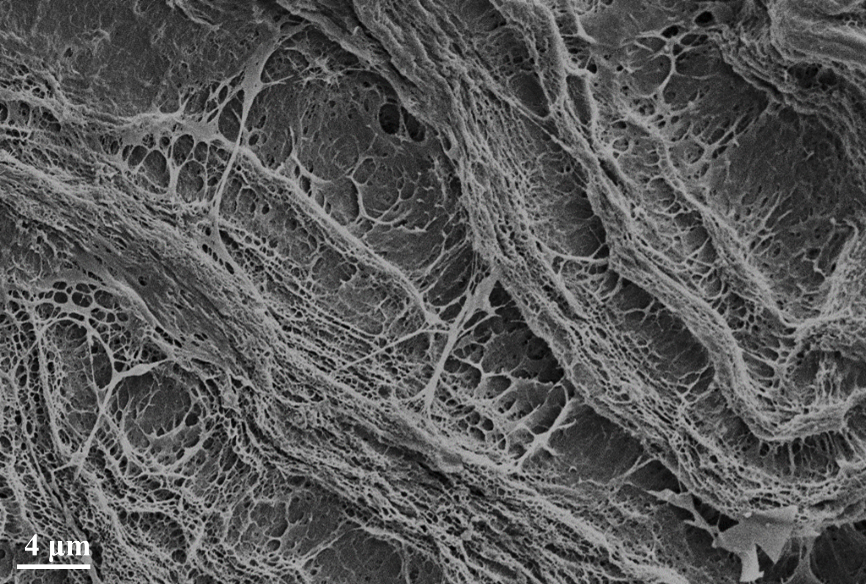


Fig. S5 SEM images of FE


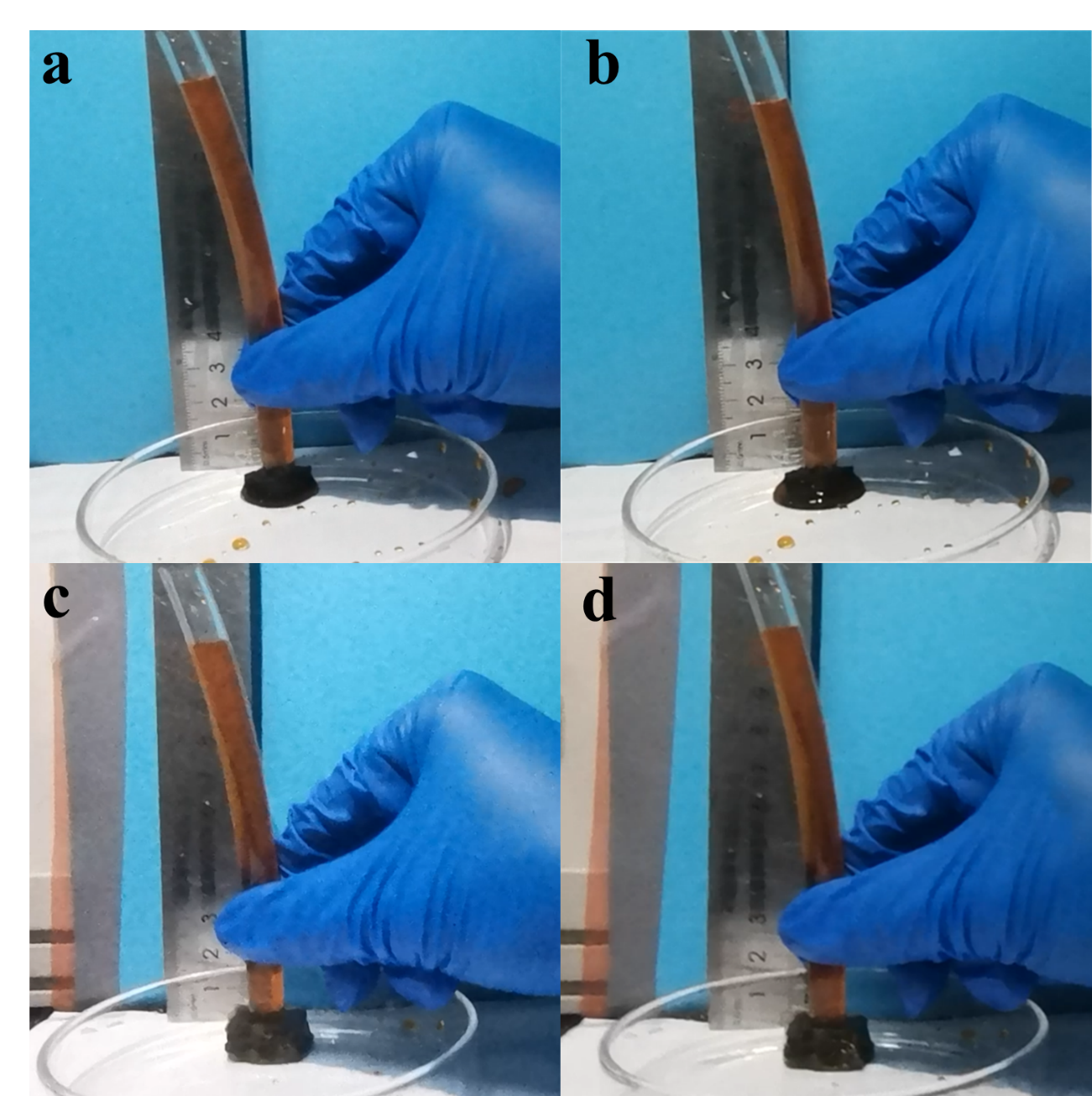


Fig. S6 Images of SCE-5 (a, b) and FE (c, d) before and after being pressed with a tube containing deionized water dyed with methyl orange.

After 30 s, the water level in the tube on FE keeps unchanged, and no liquid flows out from the bottom of FE. By contrast, the water level of the tube on SCE-5 gradually drops, and red liquid flows out from the bottom and side of SCE-5.


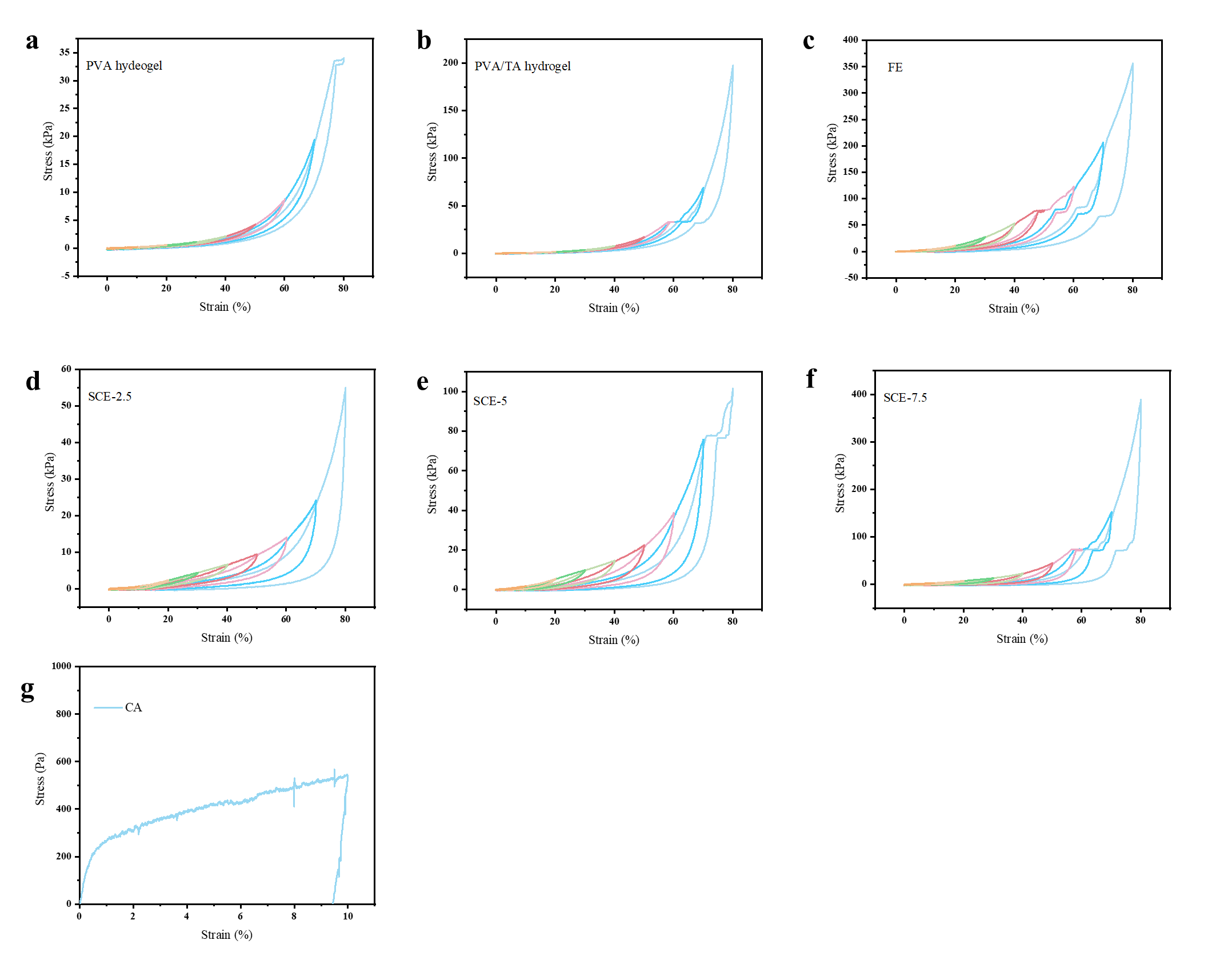


Fig. S7 the compression strain-stress curves of a series of evaporators.

Fig. S8 The full spectrum of XPS for CAH and SCE


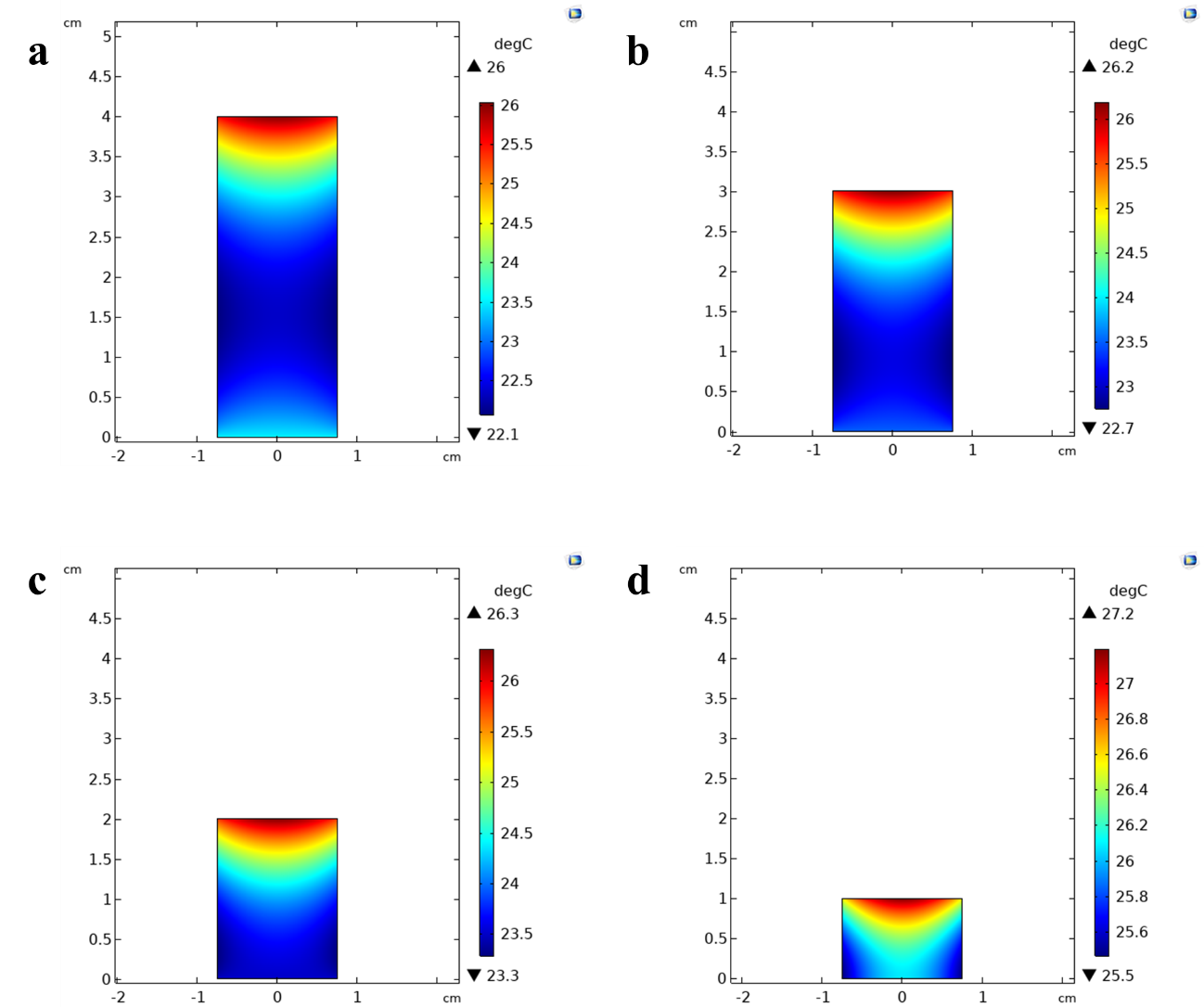


Fig. S9 Simulated temperature distribution of SCEs for H=(a) 4 cm, (b) 3 cm, (c) 2 cm and (a) 1 cm using COMSOL Multiphysics 5.5.

Fig. S10 mass change of CA with different Hs under 1 sun.


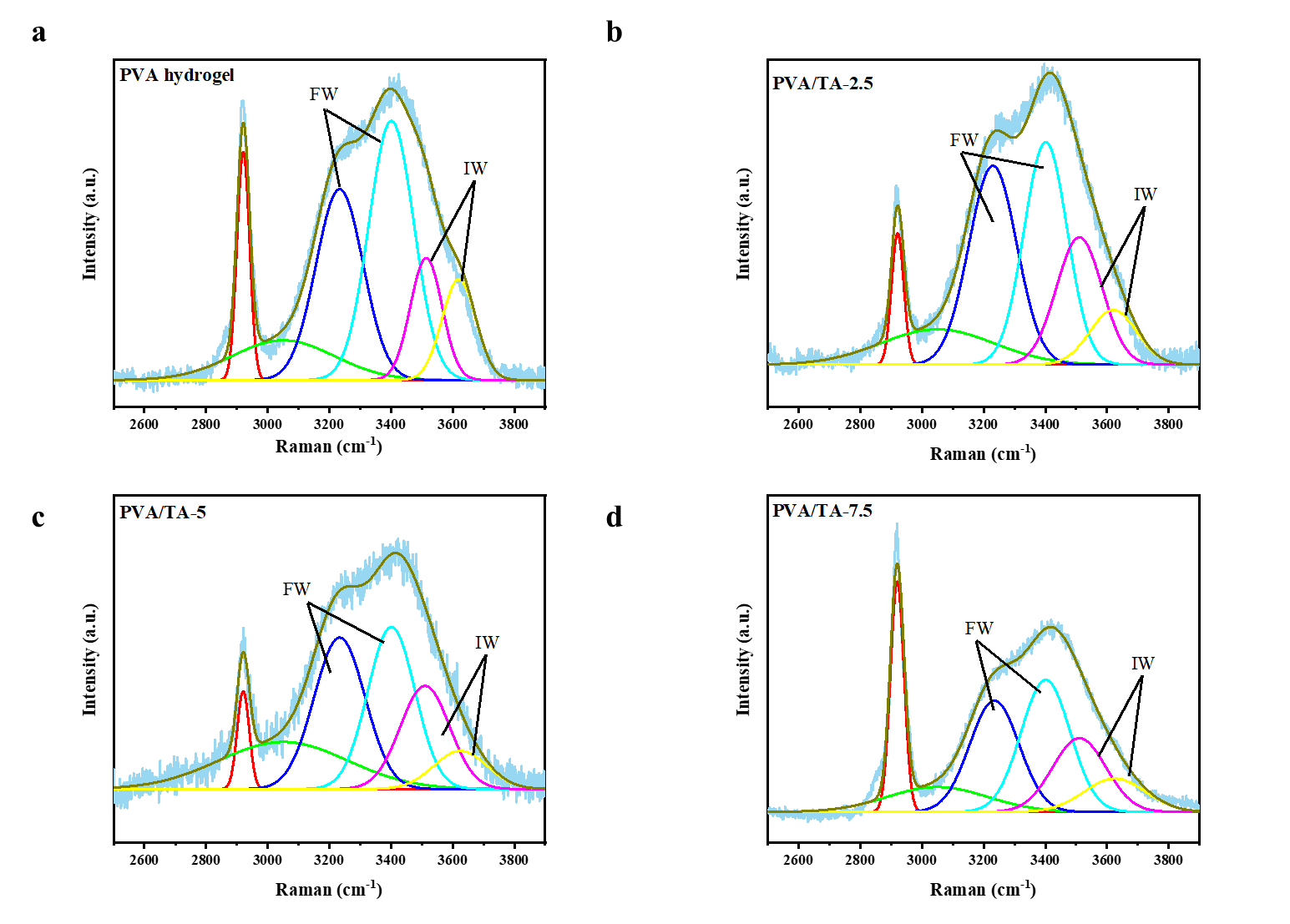


Fig. S11 Raman fitting curves of water in each evaporator.

Fig. S12 Concentrations of Li^+^ in original bulk water and in the collected condensate water form SCE-based evaporation and direct-evaporation.

1. Yan, J.; Wu, Q.; Wang, J.; Xiao, W.; Zhang, G.; Xue, H.; Gao, J., Carbon nanofiber reinforced carbon aerogels for steam generation: Synergy of solar driven interface evaporation and side wall induced natural evaporation. *J. Colloid Interface Sci.* **2023,** *641*, 1033-1042.

2. Fillet, R.; Nicolas, V.; Fierro, V.; Celzard, A., Modelling heat and mass transfer in solar evaporation systems. *International Journal of Heat and Mass Transfer* **2021,** *181*, 121852.
